# A taxonomically harmonized global dataset of wild bird hosts for avian influenza virus surveillance

**DOI:** 10.1038/s41597-025-06451-1

**Published:** 2025-12-19

**Authors:** Fanshu Du, Qiang Zhang, Yachang Cheng, Yang Liu, Weipan Lei, Lu Wang, Honglei Sun, Yipeng Sun, Jinhua Liu, Zhichao Li, Juan Pu

**Affiliations:** 1 National Key Laboratory of Veterinary Public Health and Safety, Key Laboratory for Prevention and Control of Avian Influenza and Other Major Poultry Diseases, Ministry of Agriculture and Rural Affairs, College of Veterinary Medicine, China Agricultural University, Beijing, 100193 P. R. China; 2 Institute of Geographic Sciences and Natural Resources Research, Chinese Academy of Sciences, Beijing, 100101 P. R. China; 3 University of Chinese Academy of Sciences, Beijing, 100101 P. R. China; 4 Third Institute of Oceanography, Ministry of Natural Resources, PRC, Daxue Road No. 178, Siming District, Xiamen, Fujian 361005 P. R. China; 5 State Key Laboratory of Biocontrol, School of Ecology, Sun Yat-Sen University, Shenzhen, 518107 P. R. China; 6 Key Laboratory for Biodiversity Science and Ecological Engineering, Demonstration Center for Experimental Life Sciences & Biotechnology Education, College of Life Sciences, Beijing Normal University, Beijing, 100875 P. R. China

**Keywords:** Influenza virus, Molecular ecology

## Abstract

Wild birds are key natural reservoirs and play a central role in the global spread of avian influenza viruses (AIVs). However, the absence of a standardized global list of wild bird hosts has limited comprehensive AIV risk monitoring and assessment within the One Health framework. Here, we generate a taxonomically harmonized dataset of AIV wild bird hosts, derived from 23,358 viral isolates of wild bird origin reported in the GISAID EpiFlu^TM^ database from 1973 to 2023. Host names were systematically extracted, validated, and harmonized to resolve reporting inconsistencies and unify taxonomy across records. The dataset comprises 394 wild bird species spanning 26 orders, with Anseriformes and Charadriiformes representing a substantial share of host diversity. By clarifying the global spectrum of wild bird hosts for AIVs, this dataset provides a foundation for host identification, phylogenetic annotation, and ecological trait-based analysis. Structured in machine-readable formats, it enables reproducible and large-scale, species-level studies spanning virology, epidemiology, and biodiversity.

## Background & Summary

Wild birds, particularly waterfowl of the order Anseriformes and shorebirds of Charadriiformes, are widely established as natural reservoirs of avian influenza viruses (AIVs)^1–3^. They play central roles in the intercontinental spread, genetic reassortment, and cross-species transmission of AIVs^3–10^. The rapid evolution of these viruses, driven by frequent mutation and reassortment, has led to a growing number of cross-species spillover events from wild birds to domestic poultry in recent years^6,11–13^. Some strains carried by wild birds have also acquired the ability to cross species barriers and infect a wide range of mammalian hosts, including humans^14–18^. Such cross-species transmission has triggered intercontinental outbreaks, disrupted poultry production, and raised major global public health concerns. Over the past two decades, successive waves of global AIV outbreaks have also underscored the urgent need for coordinated and sustained surveillance and mitigation strategies^8,9,14,19,20^.

Wild birds act as reservoirs, amplifiers, or bridge hosts of AIVs, depending on their traits and behaviours^6,21,22^. For instance, mallards (*Anas platyrhynchos*) likely act as key reservoirs of clade 2.3.4.4b in North America because of their high viral shedding, transmissibility, and mild clinical signs^23,24^. In contrast, ruddy turnstones (*Arenaria interpres*) can asymptomatically shed low-pathogenicity avian influenza viruses following low-dose exposure^25^. These examples illustrate the ecological heterogeneity of wild bird hosts in terms of AIV dynamics, highlighting distinct functional roles in viral maintenance, amplification, and cross-species transmission. The continuous expansion of global AIV surveillance has yielded a growing volume of viral gene sequences from wild birds^26^, providing unprecedented opportunities to analyse species-specific functional roles across different stages of viral ecology. Importantly, such insights aid in precisely identifying high-risk avian species and offer a scientific foundation for developing targeted surveillance strategies and evidence-based control measures.

Despite the accumulation of substantial host-related data in global avian influenza surveillance systems, the practical use of wild bird host information remains challenging. According to World Health Organization naming guidelines^27^, influenza virus names should follow the structure “type/host/location/strain number/year”. In practice, however, the absence of standardized conventions for host names has led data contributors to record them inconsistently, using common, scientific, or even local names, or with occasional spelling errors. Consequently, multiple variants exist for the same bird species, a problem further compounded by ongoing revisions to global avian taxonomy systems.

The GISAID EpiFlu^TM^ database^26^ provides metadata for each viral entry, including the host, collection date, and location, which are frequently used in viral ecology studies^8,10,28–32^. However, host information is often inconsistent; the ‘host entry’ field may not match the host embedded in virus names, and records are frequently labelled with broad taxonomic levels (e.g., group or family) rather than at the species level, resulting in a critical loss of resolution. While such discrepancies can be corrected manually in small datasets, large-scale studies relying on automated pipelines are particularly vulnerable to these systemic biases. These inconsistencies present a major challenge for virologists, most of whom lack formal ornithological training and may not recognize host-level errors. For example, a recent study revealed synchrony between bird migration and virus spread at the order level but was unable to resolve species-specific roles due to inconsistent host annotations and limited tracking data^10^.

To date, a globally standardized, taxonomically resolved list of wild bird hosts for AIV surveillance is lacking. This gap limits species-level ecological analysis, hinders the identification of high-risk hosts, and constrains the design of targeted surveillance strategies. Here, we present a taxonomically harmonized dataset of wild bird hosts for AIV, derived through systematic extraction and verification of host information from strain names of animal-origin influenza A virus isolates collected between 1973 and 2023 in the GISAID EpiFlu^TM^ database^26^. The curated dataset resolves inconsistencies in host labelling and provides harmonized species-level annotations, enhancing the reliability of surveillance, ecological modelling, and spillover risk assessment within the One Health framework.

## Methods

**Fig. 1:**
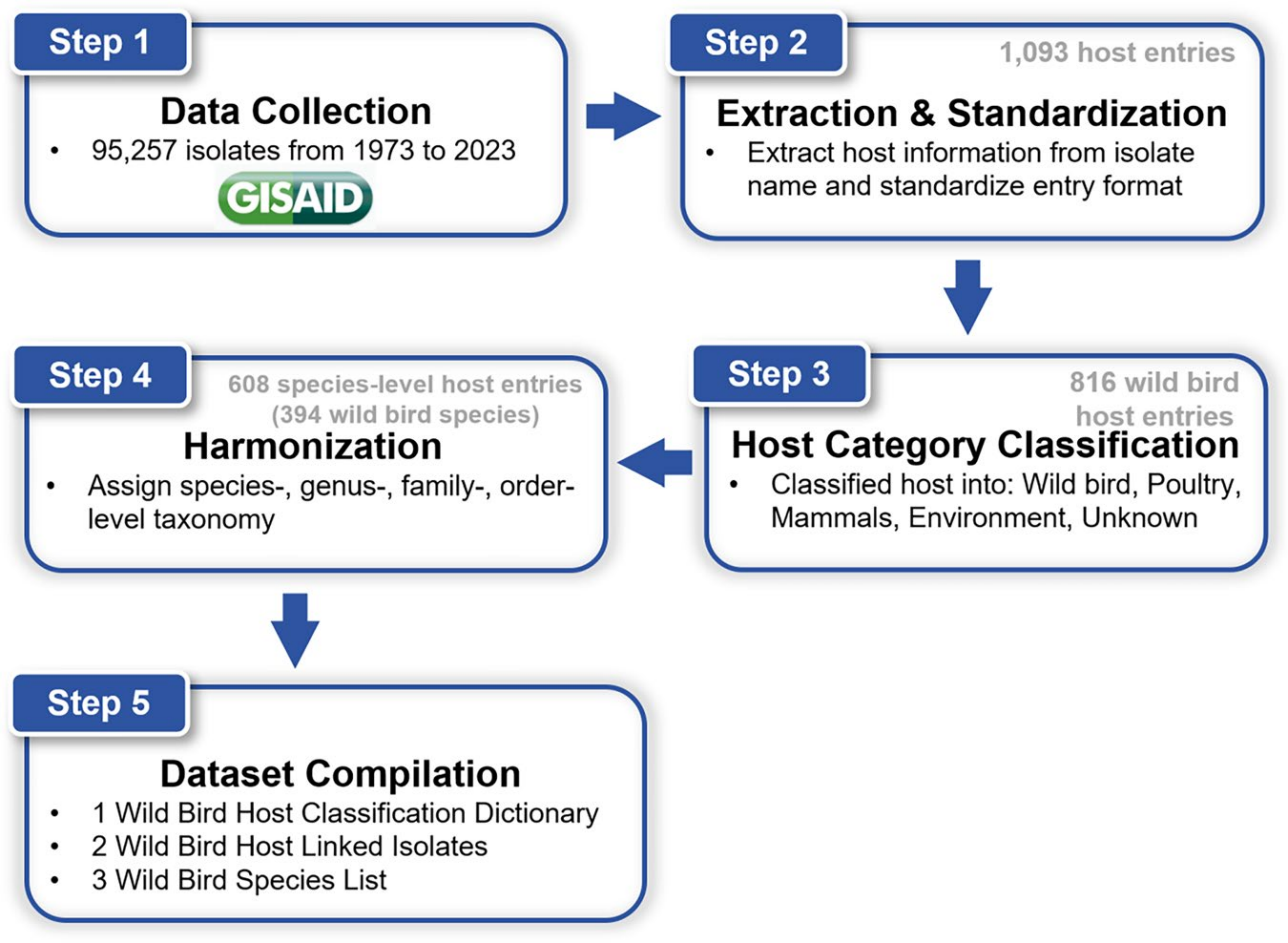
Workflow of verifying host information and for taxonomic assignment of influenza A virus isolates derived from wild birds.

The construction of the harmonized wild bird host dataset followed a five-step pipeline (Fig. 1). First, we retrieved influenza A virus isolates of animal origin from the GISAID EpiFlu^TM^ database (Step 1). Host information was then extracted from the isolate names and standardized to a consistent format (Step 2). Each entry was categorized into one of five host groups: wild birds, poultry, mammals, environment, or unknown (Step 3). Next, wild bird entries were taxonomically harmonized by assigning species-, genus-, family-, and order-level classifications on the basis of *AviList: The Global Avian Checklist (v2025)*^33^ (Step 4). Finally, we compiled a structured entry dictionary summarizing each unique host entry, aggregated species-level data, including the number of associated isolates, and annotated each virus record with complete taxonomic information (Step 5).

### Influenza virus data collection

In this study, isolate records from the GISAID EpiFlu^TM^ database^26^ were retrieved, and entries corresponding to type A influenza viruses isolated from animal hosts were selected, with collection dates ranging from January 1, 1973, to December 31, 2023. The data were downloaded on April 28, 2024. A total of 95,257 isolate records from wild birds and other animal hosts were obtained, along with associated metadata, including *Isolate_ID*, *Isolate_Name*, *Subtype*, *Location*, *Host*, and *Collection_Date*. All EPI accession numbers corresponding to the wild bird-associated isolates used in this study are provided in Avilist-Wild_Bird_Host_Linked_Isolates.csv.

### Host information extraction and standardization

Although host data are recorded in the original metadata, the isolate name often contains more precise and species-level host identifiers. For example, Table 1 presents several representative examples of mute swan (*Cygnus olor*) isolates. The original host recorded in the metadata was vague (e.g., “Swan”, “Other avian”, or “Animal”), whereas the *Isolate_Name* field included precise species-level identifiers such as “*Cygnus olor*” or “mute swan”, enabling the correction and standardization of host names. Accordingly, we extracted host terms from the *Isolate_Name* field based on the influenza virus naming format (type/host/location/isolate ID/year). To standardize the entries, all host names were converted to lowercase, hyphens and underscores were replaced with spaces, and leading/trailing whitespace was removed. This approach resulted in a dataset containing 1,093 host entries.

**Table 1:** Host information verification for Mute Swan (*Cygnus olor*) influenza virus isolates.

Isolate ID	Isolate Name	Host in Metadata	Host in Isolate Name	Host After Check
EPI_ISL_10008	A/Cygnus olor/Astrakhan/Ast05-2-2/2005	Swan	*Cygnus olor*	Mute swan
EPI_ISL_11259264	A/mute_swan/Ireland/033169_22VIR1325-14/2021	Other avian	mute swan	Mute swan
EPI_ISL_1033124	A/mute swan/Czech Republic/2600/2021	*Cygnus olor*	mute swan	Mute swan
EPI_ISL_18271512	A/mute_swan/Denmark/06339-1.01/2023-08-05	Animal	mute swan	Mute swan

### Host category classification

A total of 1,093 extracted host entries were classified into five major categories: wild birds, poultry, mammals, environment, and uncertain origin (NA). Among these, 816 entries were identified as wild bird hosts.

### Harmonization of wild bird hosts

Each of the 816 wild bird host entries was systematically evaluated for taxonomic specificity to determine the most precise identifiable rank—class, order, family, genus, or species. For entries that could be confidently assigned to the species level, both scientific and common names were recorded (Table 2). To ensure consistency and taxonomic validity, species names were harmonized according to AviList^33^, which is a collaborative global effort to produce a single current consensus taxonomy for the birds of the world, along with key information on taxonomy and nomenclature.

**Table 2:** An example of verified host records for the Tundra Swan with species and axonomic assignment.

Host Entry	Confirmed Common Name	Revised Taxonomic Assignment
bewick swan	Tundra swan	Anseriformes-Anatidae-Cygnus-C*ygnus columbianus*
bewicks swan		
Bewick’s swan		
Cygnus columbianus		

A total of 394 distinct wild bird species were identified, and for each, the scientific classification, common name, and corresponding number of virus isolates were compiled to generate a wild bird host species table. Entries with vague or generalized descriptions (e.g., “migratory bird” and “swan”) were assigned to the most specific possible higher-level taxonomic category, such as genus, family, order, or class (Table 3). The resulting classifications were used to construct a taxonomic dictionary linking all 816 wild bird entries to their corresponding scientific classification.

**Table 3:** Representative wild bird host records with non-species-level identification and closest taxonomic attribution.

Host Entry	Most Specific Assignable Taxonomic Unit	Taxonomic Level
migratory bird	Aves	Class
sparrow	Passeriformes	Order
wild duck	Anseriformes–Anatidae	Family
swan	Anseriformes–Anatidae–Cygnus	Genus

### Dataset complication

Metadata associated with wild bird-derived influenza virus isolates were further harmonized. Each isolate was matched to the scientific taxonomy and common name of its host. Additional metadata fields were normalized for further analyses. The location field was simplified by extracting the first two geographic levels (i.e., continent and country/region) and combining them into a new variable (i.e., *Continent_Place*). The *Collection_Date* field was then reduced to *Collection_Year*. The subtype field was standardized to the *HxNySubtype* format, with *HASubtype* and *NASubtype* extracted separately for subtype-specific analyses. These steps yielded a curated dataset integrating wild bird taxonomy with associated influenza virus metadata.

### Overview of the dataset

A total of 1,093 host entries were classified into two broad categories: wild bird hosts and non-wild bird hosts. Among the host entries, wild bird hosts accounted for 816 entries and 23,358 virus isolates. This included 608 entries with species-level identification, linked to 19,613 isolates, and 208 entries with vague descriptions that could not be assigned to a specific species, corresponding to 3,745 virus isolates (Table 4).

**Table 4:** Summary of host information categories and corresponding virus isolate counts.

Category	Subcategory	Number of host entries	Number of virus isolates
Wild bird host	Wild bird species	608	19,613
	Non-wild bird species	208	3,745
Non-wild bird hosts	Mammal	97	31,690
	Poultry	97	39,435
	Environment	4	40
	Not Known	79	731

**Fig. 2:**
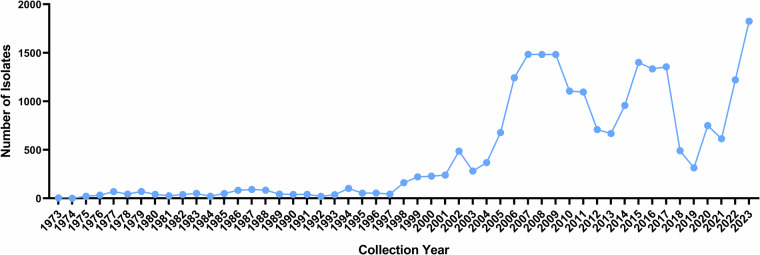
Temporal distribution of wild bird-derived influenza virus isolates.

From a temporal perspective (Fig. 2), the number of wild bird-derived influenza virus isolates increased significantly after the year 2000, indicating several distinct periods of peak activity. These include the first peak during 2006–2011, the second during 2014–2017, and the most recent peak during 2020–2023. In 2023, the number of isolates reached a record high of 1,826, the highest value across the entire observation period.

**Fig. 3:**
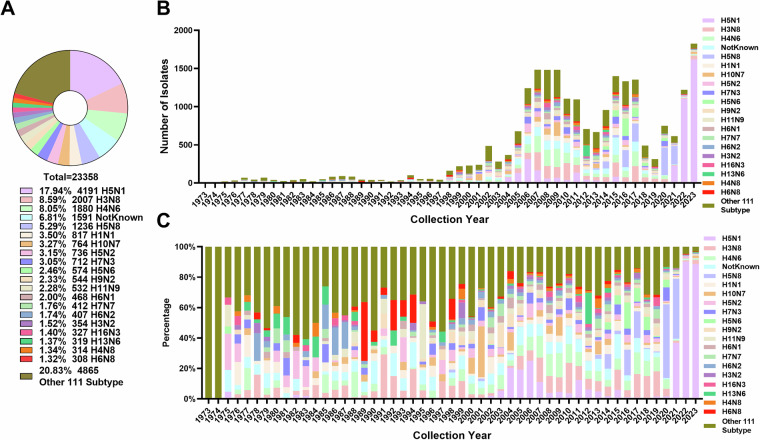
Subtype composition and temporal trends of wild bird-derived influenza virus isolates.

Moreover, the results of the subtype analysis (Fig. 3) revealed that H5N1 (17.94%), H3N8 (8.59%), and H4N6 (8.05%) were the three most frequently detected subtypes, corresponding to 4,191, 2,007, and 1,880 isolates, respectively (Fig. 3A). The annual distribution and proportional composition of different subtypes over time are shown in Fig. 3B,C, respectively. The first peak period (2006–2011) was characterized by frequent detections of H5N1, H3N8, and H4N6. During the second peak (2014–2017), H5N8 and H5N6 emerged as the dominant subtypes, with additional detections of H3N8 and H4N6. In the third peak (2020–2023), H5N1 became the predominant subtype. In the first two years of this period, H5N8 was still frequently detected, but by 2022 and 2023, more than 88% of the isolates were of the H5N1 subtype. Overall, the composition of the dominant subtypes varied notably across periods, with H5N1 emerging as the most prevalent subtype in recent years.

**Fig. 4:**
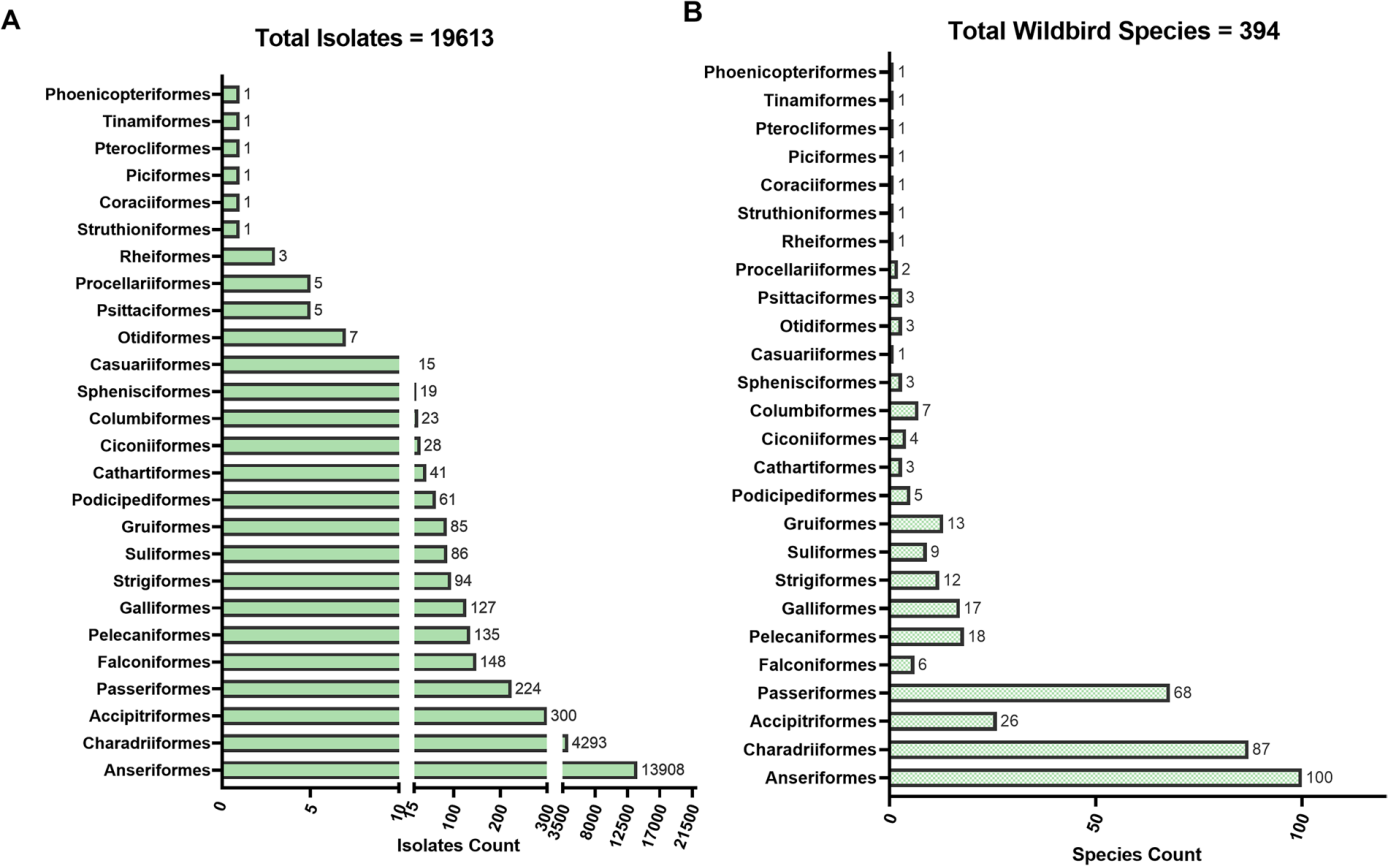
Distribution of influenza A virus isolates and host species across wild bird orders.

Among the wild bird-derived isolates, 19,613 influenza A virus isolates were identified at the species level, corresponding to 394 wild bird species from 26 bird orders. As illustrated in Fig. 4, the orders Anseriformes and Charadriiformes were predominant in both the isolates (13,908 and 4,293, respectively) and the number of species (100 and 87, respectively), emphasizing their central roles in the ecological network of influenza A viruses. In contrast, Passeriformes, despite being highly species-rich (68 species), contributed relatively few isolates (224 isolates). Accipitriformes showed the opposite pattern, with 300 virus strains identified across only 26 species. These distributional patterns highlight the differences in the ecological roles of bird orders in AIV transmission dynamics.

**Fig. 5:**
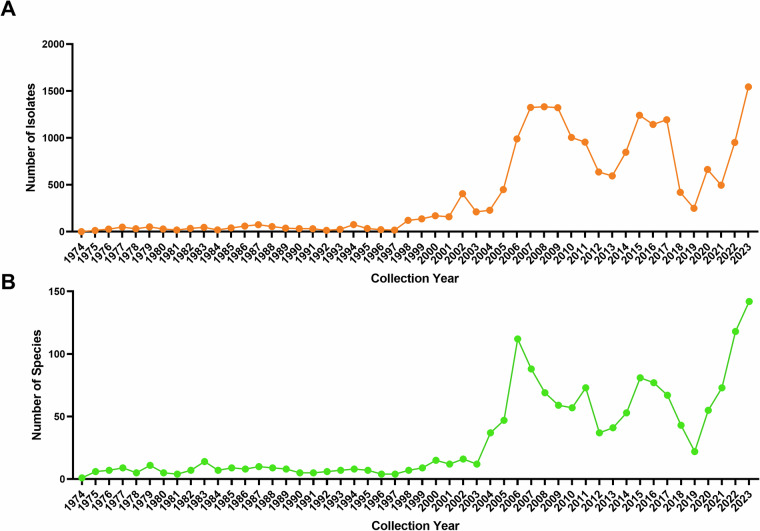
Temporal trends in wild bird species-derived influenza virus isolates and host species diversity.

The annual number of virus isolates remained low before 2000 but increased sharply thereafter, with three distinct peaks observed during 2006–2011, 2014–2017, and 2020–2023, reaching a maximum in 2023 (Fig. 5). The annual number of wild bird species identified as virus hosts exhibited a pattern largely consistent with the isolate counts. The increase in host species diversity during epidemic peaks suggests a broader ecological involvement of wild bird species in viral dissemination over time.

We further compared key fields in our dataset with those available in the GISAID database (Table 5). While both resources provide essential metadata such as isolate identifiers, host information, and collection details, our dataset offers a broader range of standardized attributes, including harmonized subtype classifications, continent- and year-level fields, host categories, and a complete taxonomic hierarchy (from order to species level) linked to AviList identifiers. This comparison highlights the unique contributions of our dataset, particularly its taxonomic harmonization across all known avian influenza host species and its global, annual coverage over two decades. By situating our dataset within the context of existing resources, we demonstrate more clearly the added value it provides for avian influenza surveillance and ecological studies.

**Table 5:** Comparison of key data fields between our dataset and the GISAID database.

Fields	Our data	GISAID	Fields	Our data	GISAID
Isolate_Id	✓	✓	Strain_Host	✓	×
Isolate_Name	✓	✓	Strain_Host_Entry	✓	×
Location	✓	✓	Scientific_name	✓	×
Host	✓	✓	English_name_AviList	✓	×
Collection_Date	✓	✓	Order	✓	×
HxNysubtype	✓	×	Family	✓	×
HAsubtype	✓	×	Genus	✓	×
NAsubtype	✓	×	Species	✓	×
Continent_Place	✓	×	Taxon_rank	✓	×
Collection_Year	✓	×	AvibaseID	✓	×
Host_Category	✓	×			

## Data Records

All the datasets generated in this study are publicly available on Zenodo in structured and machine-readable formats (.csv) at 10.5281/zenodo.15970977^34^, and each serves a distinct function. Missing or unavailable information is indicated as “NA”.

1. ***Avilist-Wild_Bird_Host_Classification_Dictionary.csv***

This file provides a comprehensive dictionary of wild bird hosts of influenza viruses. It contains 816 wild bird host entries. Each entry is annotated with detailed taxonomic information and the corresponding common name. The dataset includes eight columns:

- *Strain_Host_Entry*: host information parsed from the isolate name and formatted into a standardized entry for subsequent harmonization;
- *HostCategory*: the broad host category (all entries are wild birds);
- *Scientific_name*: the scientific name of the host species;
- *English_name_AviList*: the English name for the species according to AviList;
- *Order-Family-Genus-Species*: the full taxonomic hierarchy (order, family, genus, species) corresponding to each entry based on AviList; missing ranks are recorded as “NA”;
- *Taxon_rank*: the most specific taxonomic rank of classification achieved for each entry, including class, order, family, genus, and species;
- *AvibaseID*: the unique alphanumeric code in AviList that is a stable taxonomic identifier related to the taxonomic concept, independent of nomenclature;
- *Isolate_Count_Sum*: the number of influenza virus isolates linked to each host entry.

2. ***Avilist-Wild_Bird_Host_Linked_Isolates.csv***

The metadata table links 23,358 influenza virus isolates (based on EPI identifiers) of wild bird origin with host taxonomic information. Each entry includes associated metadata and the corresponding scientific classification and common name of the host. This file contains the following columns:

- *Isolate_Id and Isolate*_Name: the unique identifier and full virus strain names;
- Subtype, HxNysubtype, HAsubtype, and NAsubtype: the subtype information in both combined (HxNy) and separate (HA/NA) forms;
- *Location*: the original geographic location from the GISAID metadata;
- *Continent_Place*: the harmonized geographic label combining the first two levels of location (continent and country/region);
- *Host*: the original host annotation from the GISAID metadata;
- Collection_Date and Collection_Year: the sampling date and the extracted year of collection;
- *Strain_Host*: the original host name parsed from the virus isolate name;
- *Strain_Host*_Entry: the host information parsed from the isolate name and formatted into a standardized entry for subsequent harmonization;
- *Host_Category*: the broad host group; only entries classified as wild birds are included in this dataset;
- *Scientific_name*: the scientific name of the host species; entries not classified to the species level are marked as “NA”;
- *English_name_AviList*: the common name according to AviList; entries not classified to the species level are marked as “NA”;
- *Order-Family-Genus-Species*: the full taxonomic hierarchy (order, family, genus, and species) corresponding to each entry, based on AviList; missing ranks are recorded as “NA”;
- *Taxon_rank*: the taxonomic rank, including class, order, family, genus, and species;
- *AvibaseID*: the unique alphanumeric code in AviList that is a stable taxonomic identifier related to the taxonomic concept, independent of nomenclature.

3. ***Avilist-Wild_Bird_394_Species_List.csv***

This file lists 394 wild bird species identified as confirmed hosts of influenza viruses. It includes four columns:

- *Order-Family-Genus-Species*: the full taxonomic hierarchy (order, family, genus, and species) corresponding to each entry, based on *AviList v1.0 (2025)*;
- *English_name_AviList*: the English common name of each species;
- *AvibaseID*: the unique alphanumeric code in *AviList v1.0 (2025)* that is a stable taxonomic identifier;
- *Isolate_Count_Sum*: the total number of virus isolates associated with each species.

## Technical Validation

All host entries were manually curated to ensure classification. Data extraction and initial categorization were conducted by F.D. following predefined rules based on taxonomic hierarchy and host naming patterns. A parallel round of independent filtering was performed by Q.Z. to crosscheck the preliminary classifications and reduce subjectivity. The entire dataset was then reviewed by Y.C., Y.L., W.L., and Y.W.—four avian taxonomy experts with specialized knowledge in wild bird systematics—to validate species assignments and resolve ambiguous cases. This multistep protocol ensured the accuracy, consistency, and objectivity of host classification throughout the dataset.

## Usage Notes

This dataset is broadly applicable to host-based influenza virus research. The file “*Avilist-Wild_Bird Host Classification Dictionary.csv*” provides a standardized reference for wild bird hosts of influenza viruses. It can be used to extract host information from virus isolate records and to retrieve matched taxonomic classifications. The reference usage code is available at https://github.com/gogofxd/InfluenzaWildBirdHost. The file “*Avilist Wild_Bird_Host_Linked_Isolates.csv*” contains metadata for all wild bird-derived influenza virus isolates reported in GISAID up to 2023. These records have been taxonomically annotated and can be applied directly in host-related analyses without additional classification. The file “*Avilist-Wild_Bird_Species_List.csv*” provides a reference list of wild bird species for which virus isolates have been reported. It can be used directly in ecological or phylogenetic analyses and may serve as a harmonized source for host name annotations in future influenza virus sequence studies.

However, several limitations should be considered when using this dataset: (1) Unidentified or newly emerging host species: As virus surveillance continues to expand geographically and temporally, additional wild bird species may be identified as hosts of AIV. Our current dataset includes all available avian host records from GISAID at the time of compilation, but newly reported hosts may appear in the future. To address this, we will update the dataset every six months using the same workflow described in this study. In addition, because our methods and classification framework are fully transparent and openly available, users can also independently apply them to incorporate new species records and update the classification using authoritative taxonomic references such as AviList. (2) Taxonomic subjectivity and system revisions: Although the taxonomic assignments were manually curated using authoritative databases, some level of subjectivity remains, particularly for entries that could not be resolved to the species level. In addition, avian classification systems are periodically revised, and the scientific names used in this dataset may become outdated or inconsistent with future versions of bird taxonomy. (3) Database alignment and cross-database integration: The dataset and accompanying code are aligned to the GISAID EpiFlu^TM^ database. While the integration of records from other repositories (e.g., GenBank and IRD) may require adjustments to accommodate differences in field names, formats, and curation conventions, the influenza virus naming system adopted in this study is standardized across databases. Because our workflow relies on these naming conventions, it is inherently transferable and can be applied to other data sources with only minimal modifications. We selected GISAID as the primary source because it provides the most representative and comprehensive collection of avian influenza sequence records, as supported by large-scale analyses^8,35,36^. (4) Uneven temporal and geographic surveillance: The dataset is derived entirely from the GISAID EpiFlu^TM^ database, and surveillance efforts vary across regions and years; representativeness bias may thus arise in the apparent spatiotemporal distribution of AIVs. We incorporated all global records available in GISAID up to 2024, recognizing a species as a host if at least one infection was documented. Therefore, while uneven surveillance may affect spatial and temporal representation, it has minimal impact on the comprehensive identification of host bird species. (5) Behavioural and migratory traits: Although such traits are important for shaping AIV transmission dynamics, they were not included in the present dataset, as our primary goal was to establish a standardized and taxonomically harmonized reference of wild bird hosts. Nevertheless, the dataset provides a flexible foundation to which species-specific behavioural and migratory information can be incorporated in future research, thereby enabling broader ecological and epidemiological applications.

## Data Availability

The dataset associated with this study is publicly avaiable on Zenodo: https://zenodo.org/records/15970977. All data are provided in CSV format. Further details regarding the dataset structure and variable descriptions are avaiable in the Data Records section.

## Code availability

The code for the study can be accessed at https://github.com/gogofxd/InfluenzaWildBirdHost.
